# Rethinking a hybrid malaria chemoprevention delivery strategy for children in sub-perennial settings: a modelling study integrating age- and seasonally-targeted delivery

**DOI:** 10.1186/s12936-025-05630-0

**Published:** 2025-11-21

**Authors:** Swapnoleena Sen, David Schellenberg, Melissa A. Penny

**Affiliations:** 1 Swiss Tropical and Public Health Institute, Allschwil, Switzerland; 2 University of Basel, Basel, Switzerland; 3 London School of Hygiene and Tropical Medicine, London, UK; 4 The Kids Research Institute Australia, Nedlands, WA Australia; 5 Centre for Child Health Research, The University of Western Australia, Crawley, WA Australia

**Keywords:** *Plasmodium falciparum* malaria, Chemoprevention, Post-intervention effects, Mathematical model, Sub-perennial transmission, Malaria seasonality, Public health policy

## Abstract

**Background:**

The World Health Organization recommends perennial malaria chemoprevention (PMC), generally using sulfadoxine-pyrimethamine (SP) to children at high risk of severe *Plasmodium falciparum* malaria. Currently, PMC is given up to age two in perennial transmission settings. However, no recommendation exists for perennial settings with seasonal variation in transmission intensity, recently categorized as ‘sub-perennial’. Tailored chemoprevention strategies are needed to protect children during seasons and ages of highest malaria risk. The seasonal dimension must adequately cover seasonally increased risk periods, alongside interventions that address year-round, lower intensity transmission. This study proposes a hybrid malaria chemoprevention (HMC) strategy, integrating two delivery components: (1) existing PMC, and (2) additional monthly SP doses during the higher-risk rainy season, ensuring a one-month gap between any two doses.

**Methods:**

Using a validated individual-based malaria model combined with pharmacological models of drug action (OpenMalaria), the potential public health impact of the proposed HMC (for children 03–24 months), and an age-expanded HMC (referred to as HMC + , for children 03–36 months), under different drug sensitivity, coverage, and prevalence (5–70%) assumptions were examined.

**Results:**

The models predicted higher efficacy of HMC and HMC + compared to PMC alone in children under age three, estimating HMC and HMC + provide 2.1 (1.6–2.6) (median (interquartile range)) and 2.9 (2.2–3.6) times (relative fold increase in burden averted) against clinical, and 2.0 (0.6–3.4) and 3.3 (0.8–5.8) against severe cases, respectively. This led to a median protective efficacy of 31.8% (25.4–38.2%), 44.9% (36.9–52.9%) against clinical, and 16.1% (7.0–25.2%), 26.4% (14.4–38.4%) against severe cases by HMC and HMC + , respectively, across the prevalence, drug sensitivity, and coverage assumptions. Under model assumptions, the results indicated a positive net impact for children under five years of age, outweighing the limited potential for delayed malaria.

**Conclusion:**

Substantially increased public health benefits might be achieved by adding seasonally-targeted chemoprevention to current PMC in sub-perennial malaria transmission settings. Effectiveness-implementation studies will be crucial to generate empirical evidence of public health impact including on the disease burden averted, safety, and cost-effectiveness of the hybrid approach. Such studies should also explore determinants of implementation success including operational feasibility, coverage, and acceptability of proposed dosing strategies to inform deployment decisions.

**Supplementary Information:**

The online version contains supplementary material available at 10.1186/s12936-025-05630-0.

## Background

Traditionally, the malaria community has thought in terms of ‘seasonal malaria’, with intense transmissions for three to five months per year and ‘perennial transmission’, which implies malaria is transmitted year-round fairly constantly. Currently, the World Health Organization (WHO) guidelines for malaria chemoprevention in children target these two endemic settings through contextual delivery strategies [1]. Perennial malaria chemoprevention (PMC) consists of administering repeated antimalarial treatment with sulfadoxine-pyrimethamine (SP) to young children (typically 03–24 months of age) in perennial *Plasmodium falciparum* malaria transmission settings. The age-targeted dosing time points are encouraged to align with local Expanded Programme on Immunization (EPI) touchpoints. On the other hand, seasonal malaria chemoprevention (SMC) consists of three to five cycles of antimalarial treatment at least one month apart, usually to children under five years, administered during the peak transmission season in seasonal setting through fixed-point or door-to-door delivery [1–3].

However, many transmission settings do not fit into either the strictly seasonal or perfectly perennial. Most such settings have year-round transmission with seasonal variation in transmission intensity, leading to substantially higher transmission during rainy seasons [4] (such as, between December and April in parts of Mozambique [5]). Recognizing this, the WHO adopted a relatively new ‘sub-perennial’ malaria seasonality concept in 2021 [6] However, to date, there are no chemoprevention recommendations specific to these areas [1] and particularly limited consideration to address how much seasonal variation warrants prioritising seasonally-targeted interventions. As such, SMC is usually not implemented in sub-perennial transmission settings, and PMC provides reduced protection if dosing does not coincide with the peak rainfall and parasite transmission timings [7].

A PubMed search conducted between 2000 and 2024, with search term “malaria” AND “sub-perennial” OR “subperennial”, found no published study that explored any antimalarial strategy for such settings. However, additional searches for perennial settings found results from one clinical study [8], a secondary analysis utilizing former intermittent preventive treatment in infants (IPTi) trial data [9], and one modelling [10] study that indicated promising potential benefits by administering seasonally-targeted monthly SP doses in children up to 24 months in settings now defined as sub-perennial. Up to three-fold greater efficacy was observed [7, 9] for children who received their first two doses during the wet season compared to age-targeted dosing linked with the EPI schedule (75.2% vs 24.8% against clinical malaria). Similarly, higher efficacy per dose was predicted with monthly doses of SP in infants during the wet season only (26% at 24 months of age) compared with age-targeted IPTi-SP[10]. The efficacy further increased with seasonally targeted SP’s age-expansion up to 24 months (52% by 24 months of age). However, there are likely challenges to setting up and delivering seasonally-targeted SP [1], and currently, no such strategy is implemented.

Relying only on seasonally-targeted SP for the newly classified sub-perennial settings may hinder the adoption of either age-targeted SP (PMC) or the seasonally-targeted SP. In addition, research is needed to enhance PMC’s public health impact, including the benefit, cost, and coverage if any alternative regimen is needed to fit for different epidemiological or clinical contexts [1] Thus, a new chemoprevention schedule was explored to address these gaps via modelling. This study proposes that there is potential to reduce the childhood malaria burden by implementing a hybrid malaria chemoprevention (HMC) approach in sub-perennial settings by combining PMC administered throughout the year using the existing EPI platform and an additional four seasonally-targeted SP doses every year (likely through alternative delivery channels or by strengthening the capacity of EPI). This concept was assessed by modelling the proposed HMC for children 03–24 months, consistent with the typical PMC age range [1]. However, the risk of severe malaria remains high up to 36 months [1, 11], particularly in settings with increasing seasonality [11] and additional EPI touch points occur in this age range (such as for booster doses of diphtheria and tetanus vaccines and the second dose of the measles vaccine) [1, 12, 13]. Thus, an age-expanded HMC (referred here as HMC +, for children 03–36 months) was also modelled, including two more age-targeted dosing events, and another round of seasonally-targeted dosing in the third year of life. Furthermore, it is crucial to monitor post-intervention effects when designing or expanding time-limited antimalarial intervention targeted for young children to estimate any potential interference with natural immunity acquisition [14] Therefore, any risk of delayed malaria and net programme benefit were assessed for HMC and HMC +, in children up to five years of age.

Updating or designing new chemoprevention strategies requires careful planning, sustainable commitment, and communication of benefits to multiple stakeholders, including communities and families and caregivers of children. While this is primarily a mathematical modelling study, possible translation to public health practice was additionally explored in light of implementation research investigation [15, 16]. Although preliminary, this study sought to support more formal community participation and any pilot implementation study design, grounded in an understanding of the proposed intervention’s context [15] and to facilitate engagement of multidisciplinary research around a much-needed chemoprevention strategy. Altogether, this model-driven commentary aims to spur dialogue based on quantitative evidence from representative scenarios and calls for generating empirical evidence on the safety, impact, feasibility, costs and acceptability of the proposed chemoprevention for protecting underserved children in sub-perennial settings.

## Methods

### Brief description of the model

The impact of chemoprevention strategies was estimated using an open-source, individual-based stochastic model of *P. falciparum* malaria epidemiology (OpenMalaria, https://github.com/SwissTPH/openmalaria/wiki) [17, 18]. Different model variants describe varying assumptions of malaria pathophysiology, within-host parasite dynamics, the effects of comorbidity, heterogeneity of anti-malarial immunity acquisition and its decay. All models have been fitted to field data across sub-Saharan Africa, as described previously [17, 19] (supplementary Sect. 1.1, Table S1). A model variant embedding within-host parasite dynamics and drug PK/PD models was utilized [19]. Varying drug sensitivity to SP was modelled by specifying different thresholds for the half-maximal effective concentration (EC50): either full sensitivity against wild-type *P. falciparum*, or partial resistance against prevalent quadruple mutant genotypes (dhfr-51I, dhfr-59A, dhfr-108A, and dhps-437G in *Pfdhfr* and *Pfdhps* genes) [20, 21].

### Simulation scenarios

**Fig. 1:**
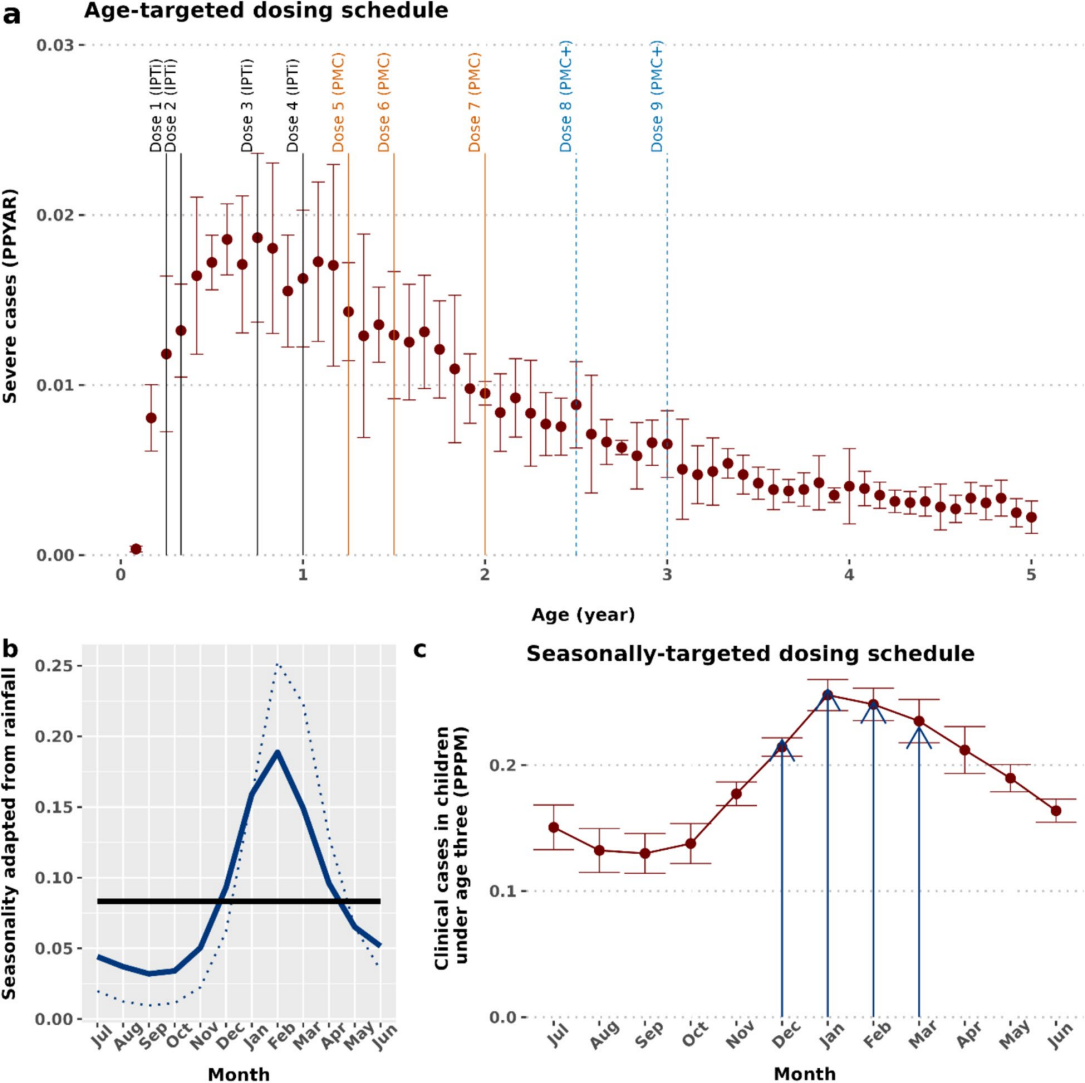
Proposed hybrid malaria chemoprevention with sulfadoxine-pyrimethamine (SP). The top panel **a** depicts PMC and PMC + dose schedule based on the age-pattern of severe malaria (median and interquartile range), and potential contacts with routine expanded programme on immunization. Four SP doses at 03, 04, 09 and 12 months of age (as per former IPTi, and also given for current PMC and for an age-expanded PMC +) is depicted by the solid vertical black line, three SP doses at 15, 18 and 24 months of age (as per current PMC) are shown by the solid orange lines, and two additional SP doses at 30 and 36 months of age (as per age-expanded PMC +) are depicted by the light blue dashed lines. The bottom left panel **b** shows an example of a seasonality pattern (reflecting percentage of average yearly rainfall distributed across months). The solid black line denotes perfect perennial transmission, dotted blue line shows seasonality pattern based on average monthly rainfall data from Mozambique, and the solid blue line indicates the modelled representative sub-perennial malaria transmission (adjusted from the Mozambique rainfall pattern). The bottom right panel **c** shows the proposed additional four seasonally-targeted dosing cycles based on the pattern of clinical malaria incidence over the year. The blue arrows show the seasonally-targeted dosing time-points. *IPTi* intermittent preventive treatment in infants, *PMC* perennial malaria chemoprevention, *PMC + * age-expanded perennial malaria chemoprevention, *PPPM* per person per month, *PPYAR* per person per year at risk

The potential public health impact was estimated, including any post-intervention effect of the proposed HMC compared to PMC alone. PMC doses aligned with age patterns of severe malaria as per current WHO recommendations (Fig. 1a), while seasonally-targeted dosing was informed by the clinical incidence pattern reflecting the seasonal distribution of disease burden (Fig. 1c). A one-month gap between any two doses was applied to avoid over-dosing [1]. Four SP dosing schedules were modelled under different drug sensitivity and chemoprevention coverage assumptions (Table 1): (i) PMC (total seven EPI-linked SP doses spread between 03 and 24 months of age) [22, 23] (ii) PMC + (total nine EPI-linked SP doses spread between 03 and 36 months of age) [22, 23] (iii) HMC (PMC, plus four monthly doses during the wet season for children 03–24 months) and (iv) HMC + (PMC +, plus four monthly doses during the wet season for children 03–36 months).

**Table 1:** Overview of the proposed hybrid malaria chemoprevention in children

Variable	Description	Value
Seasonality (transmission pattern)	Distribution of malaria incidence throughout the year, correlated with average rainfall and temperature	Representative sub-perennial [5] (*adapted from rainfall pattern in Mozambique)* Archetypal, constant perennial
*Plasmodium falciparum* prevalence (*Pf*PR_2–10_)	Test-positive patent malaria cases among 2–10-year-olds using microscopy diagnostic	*Pf*PR_2–10_ calculated as model output based on patient infection
Transmission intensity	Entomological inoculation rate (EIR), interpreted as the number of infectious mosquito bites received yearly by a human host Additionally, equivalent *Pf*PR_2–10_ values across all setting characteristics are indicated	4, 8, 16, 32, 64, 128, 256, 512, 1024 (*Pf*PR_2–10_%*:* 13, 19, 28, 37, 44, 50, 54, 57, 58)
Chemoprevention dosing schedule	Dosing timing for combining PMC or PMC + with additional seasonally-targeted SP as per WHO recommendations: the current WHO guidelines allow flexibility in terms of selecting number of doses to best suit the context, and additionally recommends at least a one-month gap between consecutive doses[1]	**HMC** **PMC**: Seven SP doses at 3, 4, 9, 12, 15, 18, 24 months of age [22, 23, 40], plus **seasonally-targeted SP**: four monthly doses in December to March (age 03–24 months) **HMC +** **PMC +**: Nine SP doses at 3, 4, 9, 12, 15, 18, 24, 30, 36 months of age[23], plus **seasonally-targeted SP**: four monthly doses in December to March (age 03–36 months)
Chemoprevention coverage (%)	Proportion of eligible children receiving a chemoprevention (PMC or PMC + or HMC or HMC +) in each dosing cycle: Full coverage―modelled to estimate maximum achievable impact, and the maximum risk of post-intervention effects Reduced coverage―modelled to simulate minimum WHO vaccination targets in all district-level populations [30, 31] and results from chemoprevention implementation studies [33]	100% (full coverage setting) 80% coverage for each cycle (reduced coverage setting). This means on average less than 10% receive all hybrid SP doses (i.e. only 8.59% receive all HMC, and 5.50% receive all HMC + doses)
Case-management coverage (%)	Access to effective treatment against clinical malaria incidence, expressed as probability in a 14-day period (aligned to definitions of treatment seeking behavior for fevers from Demographic Health Surveys, implemented as a corresponding 5-day probability in the model [25])	Low: 10% Medium: 30% High: 50% Very high: 80%
Deterministic diagnostic	Microscopy diagnostics detecting parasite density above a specified threshold	40 parasites per µL of blood [42]
PK parameters for SP^2^ (one compartment model) [21, 43]	Volume of distribution (L/kg) Absorption rate constant (per day) Elimination rate constant (per day) Negligible concentration (mg/L)	0.29 12.5 0.12 0.001
PD parameters for SP^2^ (Michaelis-Menton kinetic^3^)	Maximum killing rate (per day) Slope of the effect curve Drug concentration at which half the maximum killing rate is achieved (EC50; mg/L). Impacted by parasite genotype (mutations in key genes that drive drug-sensitivity and the duration of chemoprevention)	2.3 2.1 2.4 for partially SP-resistant having duration of protection 35 days (quadruple mutation in *Pfdhfr* gene encoding dihydrofolate reductase + *Pfdhps* gene encoding dihydropteroate synthase) [9, 20]) 0.5 for SP-sensitive having duration of protection 42 days [9, 20]

During the rainy season, seasonally-targeted SP doses replaced PMC if the schedules overlapped to prevent multiple dosing. In the model, missed age-targeted doses refer to those substituted with seasonal SP. As a result, children born close to the start of the seasonal window could miss up to three scheduled PMC doses in their first year, while those in their second and third years typically missed fewer doses (maximum of two and one, respectively), depending on their age relative to the timing of the seasonal dosing campaign.

Pre-validated EC50 values were used to model SP’s prophylactic period under different drug sensitivity assumption [20, 21]**,** reducing from 42 days in SP-sensitive setting to 35 days in partially SP-resistant settings [20].

The representative sub-perennial seasonality in this study approximates the transmission pattern in Mozambique [5, 23]. The average monthly rainfall from Mozambique was adapted within OpenMalaria (https://swisstph.github.io/openmalaria/fourier) to demarcate from a strictly seasonal setting by ensuring less than 60% rainfall during a consecutive four-month period. The distribution of entomological inoculation rate (EIR) was correlated to this in the model [1–3] (Fig. 1b, supplementary Sect.  1.2, and supplementary Table S2). However, since rainfall levels may vary between perennial to sub-perennial patterns over time, archetypal perennial settings were also modelled by a uniform year-round distribution of EIR (Fig. 1b). Case management coverage values were assumed to reflect ranges observed in sub-Saharan Africa [23–25]. The combination of setting characteristics is summarized in Table 1. The simulation scenarios were a full factorial design of these characteristics with 10 stochastic realizations per scenario.

Estimation of protective efficacy, and additional burden averted by the hybrid strategies Results were reported after five years from programme rollout to capture both the impact during the intervention and for post-intervention follow-up ages [14]. The protective efficacy (at 100% coverage) or effectiveness (at reduced coverage) was calculated as the relative reduction of incidence rate in the intervention group compared to the control group (absence of PMC or HMC). The relative fold increase in burden averted by HMC or HMC + compared to PMC was calculated as the effectiveness of HMC divided by the effectiveness of PMC.

### Estimation of post-intervention effects

Any post-intervention effects including: (i) the age-pattern of clinical and severe cases per age, and (ii) net impact (measured by the cumulative incidence by age) were estimated as per the WHO recommendation[14] up to age five. Results were reported assuming full programme coverage to predict the maximum extent of potential post-intervention effects.

### Statistical analysis

The relative contribution of different setting characteristics across the parameter ranges (indicated in Table 1) on the protective effectiveness was assessed by applying multiple factorial ANOVA test using R Package CGPfunctions[26]. The contribution of individual setting characteristics after controlling for all factors was extracted from the partial eta squared values.

### Validation of SP model and impact estimates

The proposed hybrid malaria chemoprevention study follows a PMC modelling study that predicted the public health impact of a proposed age-expanded PMC schedule [23]. The likely parasite life-stage specific mode of action of SP and validation of the model estimated effect size (protective efficacy against clinical and severe malaria) against empirical data from a wide range of epidemiological and clinical settings across Africa [5, 27] was described previously in detail [23].

### Exploration of implementation study designs and determinants of outcome

The likely know-do gaps and the complexity of implementing any new chemoprevention recommendation were recognized[22]. Thus, potential implementation designs were explored to support pilot studies for assessing the utility and feasibility of the proposed HMC or HMC + schedule. An initial discussion with implementation science researchers followed by a targeted literature search [15] was carried out to identify potential study designs that could facilitate a faster transition from proposal to practice. Additionally, the consolidated framework of implementation research (CFIR) [16] was considered as a reference to list likely determinants (such as the local and broader setting characteristics and stakeholders) of implementation outcomes (supplementary Sect. 1.3 and Table S3). These factors were prospectively mapped to the proposed chemoprevention context based on the publicly available information of a recent PMC scale-up project (MULTIPLY)[28].

## Results

### Protective effectiveness

**Fig. 2:**
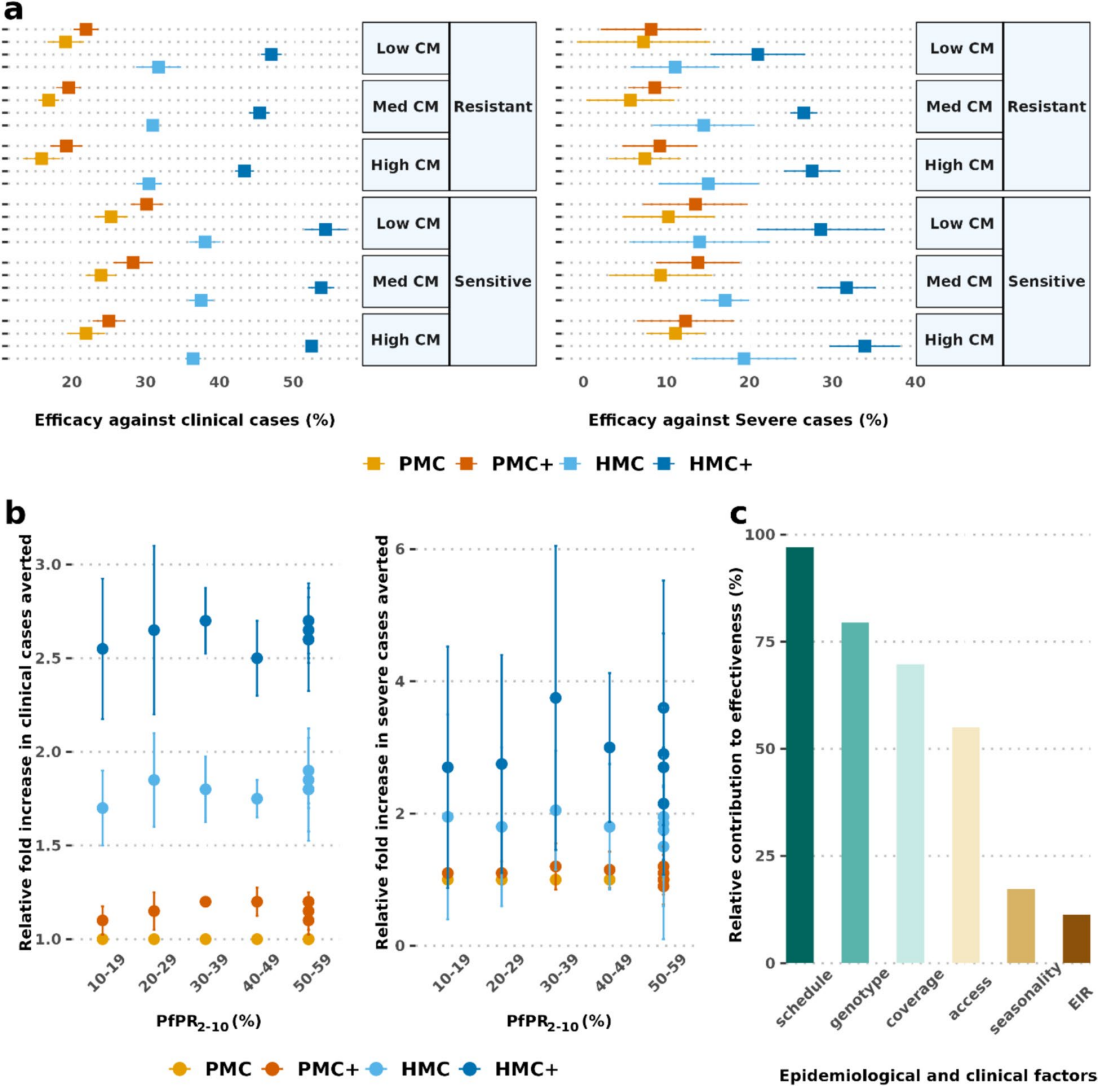
Protective efficacy and relative fold increase in burden averted by proposed hybrid dosing strategies in children under age three. Panel **a** depicts the median (interquartile range) of protective efficacy across settings. Results are shown for moderate to high transmission (baseline annual prevalence *Pf*PR_2–10_ 5–70%) and different levels of access to case management settings**. **Panel **b** depicts the relative fold increase in burden averted compared to PMC alone, while panel **c** shows the relative contribution of the setting and intervention characteristics on achievable effectiveness. The relative fold increase in burden averted here is shown for full coverage as an example of best-case scenario. *CM* access to case management, *EIR* entomological inoculation rate, *HMC* hybrid malaria chemoprevention, *HMC + * age-expanded HMC, *PMC* perennial malaria chemoprevention, *PMC + * age-expanded perennial malaria chemoprevention

Consistent with earlier studies [8–10], the results indicated substantial public health benefits of seasonally-targeted SP doses (Fig. 2a, 2b). A maximum median efficacy of 28.7% (25.1–32.3%) and 40.7% (36.2–45.2%) against clinical, and 14.4% (6.0–22.9%), and 18.1% (8.5–27.8%) against severe malaria was predicted to be achieved by HMC, and HMC + respectively, in the first three of life in partially SP-resistant setting (Fig. 2a). These values increased to 34.8% (31.5–38.1%), 48.4% (43.7–53.1%) against clinical, and 18.1% (8.5–27.8%), 29.4% (17.4–41.5%) against severe disease, under HMC and HMC +, respectively in SP-sensitive settings. Thereby, HMC and HMC + increased the impact against clinical malaria by 2.1 (1.6 −2.6) times and 2.9 (2.2–3.6) times relative to PMC alone (relative fold increase in burden averted). Corresponding values were 2.0 (0.6–3.4) and 3.3 (0.8–5.8) against severe malaria (Fig. 2b).

The statistical analysis showed that the dosing schedule will likely drive this increased protective effectiveness across epidemiological and clinical settings (Fig. 2c). Consistent with earlier studies[22, 29], the results demonstrated that the protection remained largely sustained in partially SP-resistant settings (such as quadruple dhfr-51I, dhfr-59A, dhfr-108A, and dhps-437G mutations in *Pfdhfr* and *Pfdhps* genes that reduced the prophylactic period to 35 days from 42 days in the sensitive setting)[20].

The results predicted that the enhanced impact (relative fold increase in burden averted) of hybrid chemoprevention remains high in modelled reduced coverage assumptions (Supplementary Fig. S1). These values reached 1.7 (1.2–2.2), and 2.5 (1.9–3.1) against clinical, and 1.7 (0.5–2.9) and 2.7 (0.8–4.6) against severe cases by HMC and HMC + with 80% coverage in each dosing cycle (leading to 8.59% children to receive all HMC, and 5.50% children to receive all HMC + doses). The wider uncertainty bound for relative fold changes against severe malaria compared with clinical cases, possibly reflect greater stochastic variation as the severe cases reduce to low levels in these more protected cohorts. No specific setting characteristics were associated with these results.

The statistical analysis indicated that the number of doses contributed most to increasing effectiveness, followed by coverage against clinical malaria. Coverage was more important than age-expansion against severe cases for PMC, but not for HMC.

### Overall net intervention impact

**Fig. 3:**
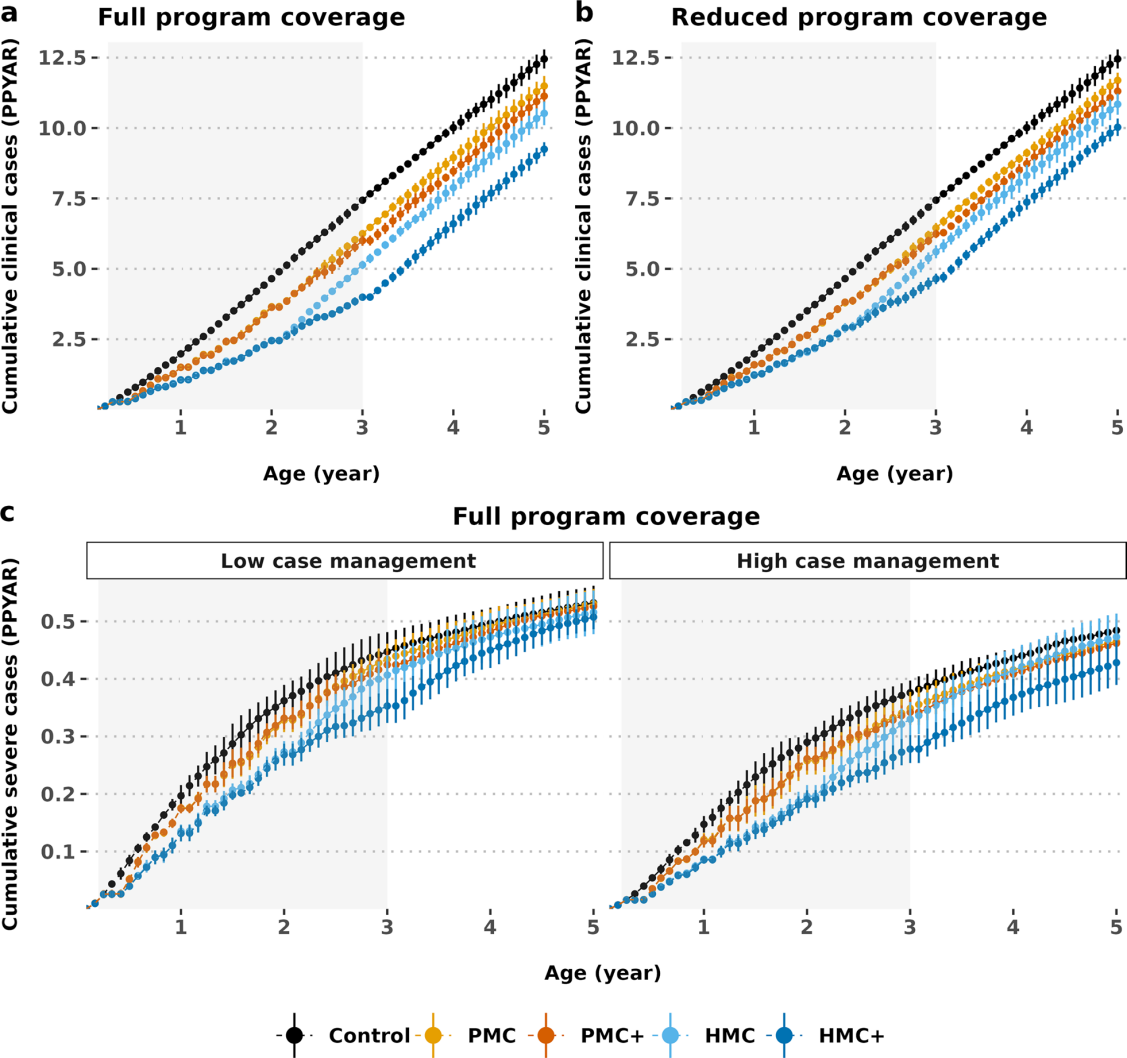
The net programme impact expressed as cumulative malaria incidence in intervention and post-intervention ages. The top panel **a** and **b** shows the net impact under full (100% in each dosing cycle of both age- and seasonally-targeted SP) vs. reduced programme coverage assumptions (80% in each dosing cycle). Results are shown in settings with *Pf*PR_2–10_ 30–39% (entomological inoculation rate 32 and 30% probability of access to case management within 14-days). The bottom panel **c** indicates the net impact in low (10% probability of seeking treatment within 14-days) vs. high access to treatment (50% probability of seeking treatment within 14-days) settings, under full chemoprevention coverage assumption. *HMC* hybrid malaria chemoprevention, *HMC + * age-expanded HMC, *PMC* perennial malaria chemoprevention, *PMC + * age-expanded perennial malaria chemoprevention, *PPYAR* Per person per year at risk

The net impact (i.e. total intervention and post-intervention effects) [14] is predicted to be positive when children up to age five were followed up. This indicates limited potential of delayed malaria for HMC and HMC + (Figs. 3a, 3b). Total impact increased in higher prevalence settings, especially when higher access to treatment was available. The net impact was also sustainable under reduced chemoprevention coverage assumption (Figs. 3c, 3 d). These results indicated that the positive net impact of proposed schedules likely outweighed the limited potential of delayed malaria, under model assumptions (supplementary Fig. S2).

### Reflection on implementation designs

**Fig. 4:**
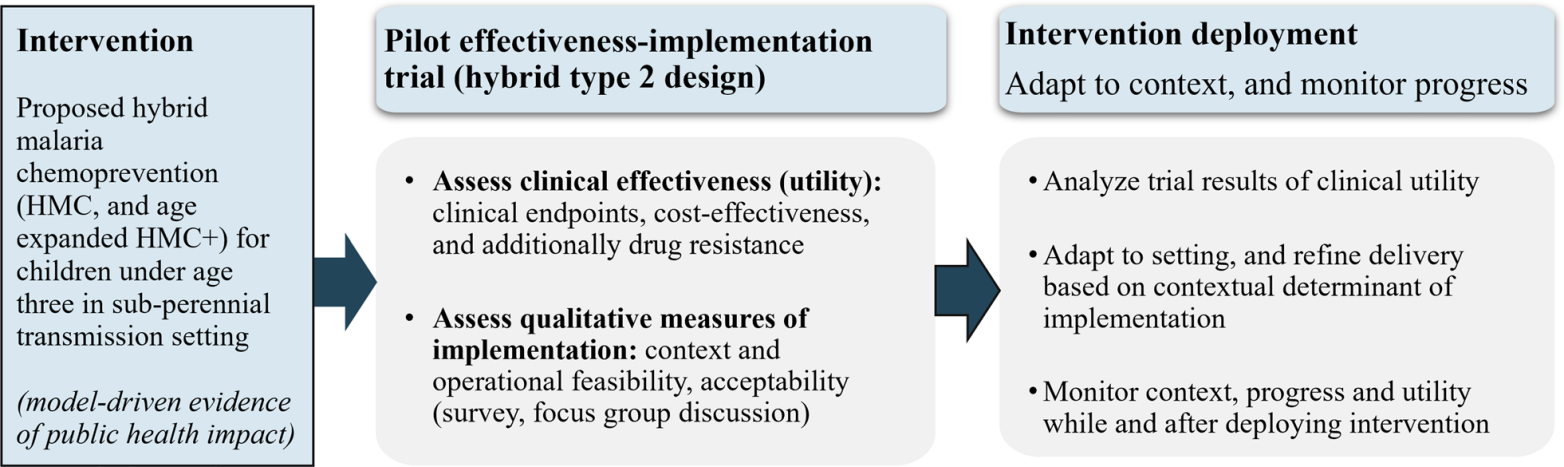
Potential implementation design for assessing the proposed hybrid chemoprevention dosing strategy in target setting

Prospective pilot studies employing hybrid study designs that simultaneously focus on assessing clinical effectiveness and implementation are likely most useful and suitable for generating empirical data on the proposed HMC and HMC + strategy [15]. As such, a hybrid type 2 effectiveness-implementation study that includes both clinical and implementation outcomes will likely generate quantitative evidence of effectiveness, cost-effectiveness, safety and any other clinical outcomes of recipients under a new chemoprevention approach. This design also includes crucial implementation study outcomes for understanding the context and determinants of programme outcomes (Fig. 4). This design is suggested for cases where data on the effectiveness of the clinical intervention is needed, as discussed later. Additionally, multiple stakeholders and factors are likely to influence these outcomes, such as national and sub-national leadership support, EPI and community health workers, cost-effectiveness, availability of case management, and funding for the hybrid deployment (supplementary Table S3).

## Discussion

Optimized chemoprevention strategies in sub-perennial malaria transmission settings have received little attention. This study aimed to initiate multi-stakeholder dialogues to support chemoprevention guidelines for these settings by providing estimates of the potential public health impact and highlighting missed opportunities to protect vulnerable children. A validated open-access malaria model [17, 21] was used to explore the potential public health benefits of complementing current PMC recommendations with additional seasonally-targeted monthly SP doses during the high-risk transmission period. Since, there is no practical experience deploying mixed malaria chemoprevention to children, this study aimed to generate preliminary, quantitative evidence to inform the planning of new dosing strategies. Furthermore, enhancing the public health impact of PMC will be beneficial in ensuring a wider uptake of this historically less utilized yet efficacious and safe intervention across recommended settings that also include sub-perennial seasonality [1]. These model-driven estimates of the potential impact, benefits or risks are anticipated to support conversations on targeted chemoprevention in these settings, as well as support plans to generate empirical safety, feasibility, and impact data through pilot implementation studies.

Overall, the model assumptions and simulated parameter ranges, including access to case management, reflect sub-Saharan African settings, where PMC is currently implemented. A proposed hybrid malaria chemoprevention (HMC) strategy to cover children up to 24 months of age was modelled, aligned with the WHO’s PMC recommendations and consistent with experience to date [1]. However, since children remain vulnerable to severe malaria, and additional EPI contacts likely remain available up to 36 months of age, an age-expanded HMC + schedule was also assessed. In addition, if these additional EPI contact points were utilized as malaria chemoprevention touchpoints, they may improve access to other vaccines or health services. Different SP-sensitivities were assumed to account for resistance and ranges of access to case management and coverage levels to predict likely public health impact across example implementation scenarios and archetypal transmission settings. The HMC + included age-expanded PMC +, which demonstrated a likely increase in net public health benefits and cost-effectiveness than current PMC [23]. The monthly SP doses were given during the rainy season, combined with PMC or PMC + throughout the year, to prevent interruption of current practices. A maximum of seven or nine PMC or PMC + doses spread over two to three years was considered, aligned with the WHO guidelines on PMC dosing frequency [1]. However, some PMC doses during the rainy season would likely be missed (i.e. replaced with seasonally-targeted doses) to ensure a one-month gap between any two SP doses. These schedules were modelled over a wide range of prevalence settings (*Pf*PR_2–10_ 5–70%) in a combination of different drug sensitivity, healthcare strength (i.e. varying access to case management), and coverage assumptions. As the mode of delivery for seasonally targeted dosing remains unknown (e.g. extended capacity within EPI, CHW delivery, or an SMC-like campaign), the same coverage level was assumed for both age- and seasonally targeted delivery. Full coverage was simulated to estimate both maximum impact and the worst-case scenario in terms of delayed malaria. In this explorative study, only one reduced coverage level was presented to illustrate the potential impact on incidence and effectiveness compared with full coverage. A per-cycle 80% coverage was assumed for both PMC and seasonally targeted SP, in line with WHO vaccination targets to reflect EPI-based PMC [30, 31] and previous SMC modelling studies [20, 32] informed by implementation study data [33]. Chemoprevention coverage is known to vary widely, from over 80% in some SMC programmes [34] to around 50% for former IPTi delivery [35]. Also, given limited PMC uptake, there are currently insufficient estimates of its per cycle coverage. Therefore, implementation studies will be crucial to understand the preferred mode of seasonally-targeted SP delivery, and to monitor cycle coverage and adherence for both age- and seasonally targeted SP.

Model estimated efficacy against clinical, and severe cases, and the likely mode of parasite life stage activity of SP was validated to empirical data from randomized controlled trials and to a recent meta-data [5, 27] as described previously [23]. Both HMC and HMC + were predicted to substantially increase protection by averting clinical and severe malaria burden compared to the current PMC alone. This is due to the greater chances of getting an SP dose, through additional seasonal dosing, during a higher-risk period. In contrast, unless a child’s age aligns with the timing of high-risk periods, PMC does not ensure adequate protection.

The results indicated increased protection and maintenance of effectiveness in modelled partially SP-resistant settings, albeit with slightly reduced total malaria averted, also in line with earlier findings [21, 22, 29]. Although, systematic reviews have confirmed that the effect of SP resistance is modest on the effectiveness of chemoprevention [29], the protection might further reduce in settings with more resistant parasite genotypes [20]. This implies that it would be prudent to monitor the evolution and spread of drug resistance by genetic biomarker surveys following continued and new chemoprevention. A recent case–control study found that a higher malaria burden may also be attributed to suboptimal drug concentration, possibly caused by missed doses rather than drug resistance [36]. Thus, alternative drug candidates must be carefully investigated to safeguard their chemoprevention benefits without compromising treatment options.

Malaria outcomes from the simulations for children older than those covered by the proposed HMC or HMC + (up to five years of age) were analysed to assess any post-intervention effects, across access to case management levels reflecting sub-Saharan African settings [23–25]. The results demonstrated a larger positive net impact (cumulative cases by age during, and in the post-intervention period) of both HMC and HMC + compared to PMC alone, thus reducing the potential delayed malaria burden. This positive net impact of HMC and HMC + remained higher than PMC alone, also for scenarios with modelled lower coverage. Notably, improved and reliable access to treatment will be necessary to ameliorate any increased risk of malaria and manage severe malaria cases after children are no longer protected by chemoprevention as is the case for any child who is no longer using an effective form of malaria prevention) [23]. This is aligned with the WHO emphasis on strengthening healthcare systems. The increased impact of these chemoprevention strategies not only eases the demand on malaria treatment but also strengthens the capacity to prevent and treat other health needs [37]. Although, these results alleviate concerns about delayed malaria, it will be important to monitor age-incidence relationships and the net impact following expanded chemoprevention programme implementation in empirical setting to confirm these model-driven findings.

To avoid potential safety concerns related to multiple dosing, the HMC or HMC + schedules were designed to restrict the number of doses each child received, ensuring a one-month gap between any consecutive age- or seasonally-targeted SP doses. However, it will be necessary to integrate stakeholder, community, and implementation perspectives in planning the timing of hybrid dosing strategies, including the total number and timing of both age- and seasonally-targeted doses to understand the feasibility, acceptability, safety, and effective implementation and communication [1].

Additionally, the uncertainties related to changing climate conditions were taken into consideration. For example, less rainfall in some years may lead to less seasonal variation in malaria transmission. Thus, the potential benefit of rolling out HMC or HMC + in both representative sub-perennial and perennial settings was examined. As anticipated, the added benefit was larger in sub-perennial settings. However, the favourable net impact of the proposed hybrid schedule was also predicted in perennial settings. These results indicate that, regardless of year-on-year rainfall and transmission uncertainty, hybrid chemoprevention delivery will likely increase the effectiveness.

Finally, it was acknowledged that conducting implementation studies in real-world scenarios is crucial to translating model-driven insights into policy or recommendations. As discussed, results from seasonally-targeted IPTi trial in Senegal [8] and modelling results built on IPTi trial data from Ghana [10] indicated a substantially larger impact of seasonally-targeted SP over a decade ago. However, no follow-up investigation or implementation occurred. Data collection for formal implementation research is beyond the scope of this modelling study; nevertheless, it aimed to address some likely next steps. This work is intended to facilitate broader discussion to ensure that new chemoprevention strategies advance beyond theoretical analysis and contribute to reducing the malaria burden in practice. To do this, possible implementation designs [15] and potential determinants of implementation for the proposed HMC [16] were presented based on literature.

Although SP is a standard of care when given as PMC, data still needs to be generated to understand the effectiveness, feasibility including cycle coverage and potential mode of delivery for the seasonally-targeted dosing, and the costs of alternative delivery strategies to support resource-allocation decisions. Therefore, hybrid type 2 effectiveness-implementation research will likely be helpful. Safety and efficacy studies may not be required given what’s known about safety & efficacy of individual doses of SP. As such, each dose protects for a certain period of time (depending on the resistance context) and that severe adverse reactions (primarily Stevens-Johnson syndrome) are idiosyncratic rather than dose-dependent [38]. Hence, evaluating effectiveness, consolidating safety and understanding impact is important. Furthermore, collecting qualitative data for the contextual determinants for tailoring delivery strategies to local contexts will be crucial to understanding how to begin rolling out any new delivery schedule. However, alternative study designs may also be considered, such as hybrid type 1 effectiveness-implementation with a primary emphasis on assessing clinical effectiveness and modest refinement to record the secondary implementation research goals. Programme success will depend on the availability of funds to deploy additional staffing at EPI facility and on the ability of community health workers to deliver both age- and seasonally-targeted dosing. Notably, expanding any prevention strategy will require comparing the cost-effectiveness of alternative approaches [23]. However, uncertainty around delivery methods and cost-per-dose estimates for seasonally targeted dosing limited economic analyses of HMC strategies in this study. It will be valuable in future work to explore cost-effectiveness again as cost data and delivery plans become available from potential implementation studies.

As with all modelling studies, these results have several limitations. First, the predictions are based on a model of blood-stage parasite-clearing activity for SP [23]. However, pyrimethamine may include liver-stage action, and any differences in immunity acquisition, assuming alternative mode of drug action dominates have not been explored [23, 32]. Also, the model does not estimate additional secondary benefits of SP beyond antimalarial effect (such as on bacterial or fungal infections) [39], which will further increase the total effect of HMC and HMC +. Second, outputs were not estimated by gender, given limited data on chemoprevention disaggregated by gender exists. Nevertheless, since the PK/PD model assumptions are consistent with earlier studies [23, 32], the broad conclusions regarding the impact of HMC and HMC + expect is expected to hold. Third, SP was modelled as used in PMC, though implementation might prefer SP-AQ during high-risk periods. However, mixing PMC with SMC would require careful consideration of different drug schedules [1]. Thus, proposed hybrid delivery schedules with SP were assessed only as a first step. SP is a relatively inexpensive, single-dose drug available within EPI and is likely to have better adherence compared to a three-day schedule. Increasing SP use may increase pressure on resistance development and spread. Thus, genomic surveillance would be prudent. Fourth, since there is currently no quantitative definition of sub-perennial transmission setting [6], a conservative threshold below strictly seasonal (i.e. less than 60% in consecutive four-month period [2, 3] was considered to define a representative sub-perennial setting. As a potential next-step for informing pilot implementation studies, the distribution of malaria cases over months was modelled based on rainfall pattern in parts of Mozambique [5, 23], where several pilot PMC (IPTi + projects) studies have been conducted [22, 23, 40]. However, results may vary in settings with different transmission profiles, such as flatter or two shorter rainy seasons [4] rather than a single prolonged one [5], reinforcing the need for a more precise definition. Finally, an implementation strategy was explored, only to initiate the discussion around possible designs.

## Conclusions

Mathematical and pharmacological modelling results demonstrated that a hybrid malaria chemoprevention approach could increase public health impact in children under three years of age, across a wide range of sub-perennial and perennial malaria transmission settings. The increase in burden averted was driven mainly by the timing of seasonally-targeted SP and the total number of doses (such as by age-expansion) rather than other setting parameters or intervention characteristics. The additional public health benefits of the proposed HMC and HMC + compared to PMC alone were realized under modelled drug sensitivity and coverage assumptions. Both HMC and HMC + demonstrated a positive net impact until age five, indicating limited delayed or age-shifted malaria burden, particularly in higher access to case management settings. However, implementation studies are needed to build on these model-driven insights and generate empirical data on clinical impact, implementation feasibility, costs and cost-effectiveness. Together, these results might support paving the way for rethinking malaria chemoprevention, informing deployment decisions[1, 41] and improving programme impact for children at high risk of severe malaria in sub-perennial transmission settings.

## Supplementary Information

Additional file 1

## Data Availability

The publicly available source code for the applied individual-based model (OpenMalaria) can be found at https://github.com/SwissTPH/openmalaria (10.5281/zenodo.10534022), including a detailed documentation at https://github.com/SwissTPH/openmalaria/wiki. The archived version of the model simulation and data analysis codes are available at (10.5281/zenodo.13804293*).* The R scripts and source data used for the production of figures presented in this paper can be found at (10.5281/zenodo.13805282).

## Abbreviations

EC50: Half-maximal effective concentration
EIR: Entomological inoculation rate
EPI: Expanded programme on immunization
HMC: Hybrid malaria chemoprevention
HMC +: Age-expanded hybrid malaria chemoprevention
IPTi: Intermittent preventive treatment in infants
PMC: Perennial malaria chemoprevention
PMC +: Age-expanded perennial malaria chemoprevention
PK/PD: Pharmacokinetic/pharmacodynamic
PPPM: Per person per month
PPYAR: Per person per year at risk
SMC: Seasonal malaria chemoprevention
SP: Sulfadoxine-pyrimethamine
*Pf*PR_2-10_: *Plasmodium falciparum* Prevalence in 2–10 year olds
WHO: World Health Organization
