# Supplementary material for: Rethinking a hybrid malaria chemoprevention delivery strategy for children in sub-perennial settings: a modelling study integrating age- and seasonally-targeted delivery
Source: Malar J. 2025 Nov 21;24:419. doi: 10.1186/s12936-025-05630-0 (PMC12639754; doi:10.1186/s12936-025-05630-0)
Supplement: Supplementary file 1 — Additional file 1 [file 12936_2025_5630_MOESM1_ESM.docx]

**Supplementary materials**

**Rethinking a hybrid malaria chemoprevention delivery strategy for children in sub-perennial settings: a modelling study integrating age- and seasonally-targeted delivery**

Swapnoleena Sen^1,2^, David Schellenberg^3^, Melissa A Penny^4,5*^

^1^Swiss Tropical and Public Health Institute, Allschwil, Switzerland

^2^University of Basel, Basel, Switzerland

^3^London School of Hygiene and Tropical Medicine, London, United Kingdom

^4^The Kids Research Institute Australia, Nedland, WA, Australia

^5^Centre for Child Health Research, The University of Western Australia, Crawley, WA, Australia

^*^Corresponding author

Email : [melissa.penny@uwa.edu.au](mailto:melissa.penny@uwa.edu.au)

# 1. Methods

## Model of malaria transmission and control (OpenMalaria)

In this model-driven commentary, the potential added public health benefit of a proposed hybrid malaria chemoprevention (HMC) strategy was explored by combining age- and seasonally-targeted sulphadoxine-pyrimethamine (SP) doses[[1](#_ENREF_1), [2](#_ENREF_2)]. An open-source, individual based model of malaria epidemiology and control, OpenMalaria[[3](#_ENREF_3), [4](#_ENREF_4)]was applied. The full range of parameters was originally calibrated to historical epidemiological data as described previously[[3-5](#_ENREF_3)], and recalibrated to the latest data[[6](#_ENREF_6)]. The source code for the model is publicly available at <https://github.com/SwissTPH/openmalaria>.

Essentially, the transmission of *Plasmodium* parasites between a mosquito vector and human host leads to infection in the human. This is simulated as a discrete, stochastic process in which the asexual blood stage parasite density in human drives the time-course and characteristics of malaria pathophysiology. The parasite density can be reduced either by naturally acquired immunity (variant-specific or variant-transcending or adaptive in the absence of any interventions), or by antimalarial treatment. The degree and duration of an intervention’s effect depends on its modelled mechanism of action, and the type of infection sub-model used (Table S1). The incidence of clinical malaria (symptomatic, but uncomplicated), severe cases (defined as per World Health Organization - WHO definition), and malaria-attributable, or all-cause mortality are tracked in each five-day timestep over the baseline (pre-intervention), intervention and post-intervention periods[[7](#_ENREF_7), [8](#_ENREF_8)].

The vector sub-model characterizes the parasite (*Plasmodium)* life cycle in mosquito vector and the probability of transmission to a human host in each (five-day) timesteps. The exposure in human is extrapolated from entomological inoculation rate (EIR) i.e. the number of infectious mosquito bites per year, and is seasonally forced by applying two-component Fourier transformation within OpenMalaria (<https://swisstph.github.io/openmalaria/fourier>). Here, a generic vectoral pattern of *Anopheles gambiae,* currently one of the most prevalent mosquito species across Africa was modelled, by simulating the entomological characteristics[[9](#_ENREF_9), [10](#_ENREF_10)].

The (OpenMalaria) model ensemble comprises of different model variants to address a variety of public health research questions. These have been developed by adapting the original model to incorporate varying assumptions about malaria transmissions, disease biology, anti-malarial immunity acquisition and decay, pharmacological effects of interventions, and impact of comorbidity on disease dynamics, among others[[11](#_ENREF_11)]. In this work, Molineux’s within-host model variant was applied, which mechanistically describes the time course of infection within each individual human host (as such, progression from asymptomatic to clinical malaria and to either severe outcome(s) or recovery)[[5](#_ENREF_5)]. This variant was used as it enables explicitly modelling population pharmacokinetic and pharmacodynamic (PK/PD) profile of chemoprevention or treatment drugs in the target population including varying assumption about drug sensitivity based on parasite genotypes. The brief description of key model components and their applications in this study are detailed in Table S1, while a full model description can be found at <https://github.com/SwissTPH/openmalaria/wiki>.

**Table S1**―**Overview of OpenMalaria model, adapted from previous publications [**[2](#_ENREF_2)**,** [12](#_ENREF_12)**,** [13](#_ENREF_13)**]**

| **Key modelled processes** | **Description and underlying assumptions** | **References** |
| --- | --- | --- |
| **Malaria disease biology and epidemiology** | | |
| Infection in individual human host | - Exposure is extrapolated from number of infectious mosquito bites over the year, specified by the entomological inoculation rate (EIR) - Seasonality pattern characterized by the monthly distribution of EIR - Correlates to availability based on age-dependent body surface area | [[3](#_ENREF_3), [4](#_ENREF_4)] |
| Progression of infection determined by asexual parasite density and acquired anti-malarial immunity | - Explicit mechanistic within-host model of asexual parasitaemia - Various natural immunity (innate, variant-specific, variant-transcending, adaptive can reduce asexual parasite density in human) acquired over multiple age-dependent infection history - Both pre-erythrocytic liver-stage and blood-stage immunity decays exponentially over time - Duration of infection follows a log-normal distribution obtained by fitting to malaria therapy dataset in the original model (collated by Marsh and Snow[[14](#_ENREF_14)]) | [[3](#_ENREF_3), [4](#_ENREF_4), [15](#_ENREF_15), [16](#_ENREF_16)] |
| Clinical cases, morbidity, mortality and anaemia | - Clinical malaria episode depends on human host parasite density and their pyrogenic threshold, which evolves over time depending on the individual exposure history - Patent malaria detected by threshold based on the type of diagnostic used (for instance, 40 parasites/µL of blood by microscopy detection was specified in this study) - Infections can be uncomplicated clinical cases, or can evolve to severe cases, based on parasite density and the pyrogenic threshold in each individual host - Severe disease can also be induced by typical age-based comorbidities - A proportion of the severe cases leads to deaths, resulting in direct malaria-related mortality - Indirect mortality classified as deaths in patients not diagnosed as malaria deaths but would not occur without a malaria exposure - All-cause mortality captures deaths resulting from both direct, indirect malaria-related causes, as well as hospitalization deaths, and thereby captures also impact of comorbidity (such as anaemia) | [[3](#_ENREF_3), [4](#_ENREF_4), [17](#_ENREF_17), [18](#_ENREF_18)] |
| **Transmission setting characteristics** | | |
| Population age structure | - Flexible and informed by health and demographic surveillance data from Tanzania | [[4](#_ENREF_4)] |
| Transmission seasonality | - Specified by monthly EIR distribution - In the absence of interventions seasonality is reproduced each year | [[15](#_ENREF_15), [19](#_ENREF_19)] |
| Transmission from infected humans to mosquitoes | - The level of infectivity depends on the density of the sexual form of parasites (gametocyte densities) present in human hosts extrapolated from blood-stage parasite densities, including a lag period - Mosquitoes become infectious follows a binomial distribution | [[3](#_ENREF_3), [4](#_ENREF_4), [20](#_ENREF_20)] |
| Entomological setting | - Mosquito lifecycle and behaviour towards human and non-human hosts (such as biting and resting) is embedded in a dynamic entomological model capturing the cycle of mosquito oviposition - Multiple vector species can be simulated simultaneously - Each variant-specific parasite has its own multiplication rate drawn from a normal distribution with mean of 16 | [[21](#_ENREF_21)] |
| **Intervention deployment characteristics** | | |
| Drugs and other therapeutic modalities | - Interventions (small molecule drugs, vaccines, monoclonal antibodies, etc.) can act at different parasite life cycle stages (either by impacting on survival and emergence of asexual liver-stage or killing blood-stage parasites, blocking transmission) | [[5](#_ENREF_5), [16](#_ENREF_16), [22](#_ENREF_22), [23](#_ENREF_23)] |
| Case management | - Modelled through a decision tree-based approach, which determines treatment implications depending on the occurrence of clinical cases - Its representation includes specification of diagnostic tests, effects of treatment, case fatality, case sequelae, and cure rates, influenced by the diagnostic threshold and access to care | [[24](#_ENREF_24)] |
| Intervention deployment characteristics (as applied to model chemoprevention,) | - In addition to treatment of clinical malaria episodes, chemoprevention interventions were deployed in the model either:  1. Continuously: to individuals at pre-specified ages over a specified time, and with specified coverage level (such as, administering perennial malaria chemoprevention) 2. Timed: at specified time points (annually or over multiple years) to targeted groups over several cycles and at specified coverage levels, typically seasonally-targeted (such as, administering seasonally-targeted sulphadoxine-pyrimethamine)  - Interventions can be deployed by enrolling individuals into cohorts and tracking cohort outcomes, facilitating clinical trial simulation | [[2](#_ENREF_2), [16](#_ENREF_16), [23](#_ENREF_23), [25](#_ENREF_25)] |
| Vector control | - Probability of infection in human host can be reduced by vector control interventions - Interventions include long-lasting insecticide-treated nets (ITN), indoor residual spraying (IRS), house screening, baited traps, mosquito repellents, and push-pull | [[21](#_ENREF_21)] |
| **Simulation characteristics and model variants** | | |
| Timestep | - Simulations are tracked for every 5-day timestep - Tracked output metrics (for instance, clinical cases) from each simulated scenario can be extracted per timestep (5 days), monthly, quarterly or yearly values | [[4](#_ENREF_4)] |
| Model variants | - Varying assumptions for immunity decay, treatment effect, and heterogeneity in transmission are captured in 14 model variants - In this study Molineux within-host model adapted including explicit PK/PD characteristic of the intervention drugs for chemoprevention drug sulphadoxine-pyrimethamine (SP), and treatment drug artemether-lumefantrine (AL) | [[2](#_ENREF_2), [5](#_ENREF_5), [16](#_ENREF_16)] |

## Scenario design

All simulations were run using ten random seeds to capture stochasticity. Trends, and dispersions for all outcome matrices were reported by calculating the median, and interquartile range respectively, as shown to be more robust to outliers from non-normal stochastic distribution[[26](#_ENREF_26)].

**Seasonality characteristics:** Two archetypal transmission settings were modelled: a representative sub-perennial (as was recorded in the replicated trial site in Manhiça, Mozambique [[2](#_ENREF_2), [27-29](#_ENREF_27)]), and constant perennial transmission. Original normalized values based on average rainfall across Mozambique resembled seasonal transmission[[1](#_ENREF_1), [28](#_ENREF_28)]. Therefore, transmission intensity was adapted by adjusting the Fourier transformation coefficient (<https://swisstph.github.io/openmalaria/fourier>, Table S2). The adjusted seasonality depicted an example of archetypal sub-perennial transmission where transmission in a consecutive five-month period was below 60%. This was done to demarcate from strictly seasonal transmission settings typically covered under seasonal malaria chemoprevention[[1](#_ENREF_1)]. The perennial transmission was modelled by uniform distribution of entomological inoculation rate over the year.

**Table S2**―**Overview of model parameters for adapting sub-perennial transmission**

| **Seasonality** | **Entomological inoculation rate rotate angle** | **Coefficient a1** | **Coefficient a2** | **Coefficient b1** | **Coefficient b2** |
| --- | --- | --- | --- | --- | --- |
| Mozambique (normalized rainfall) | 0 | 1.0414 | 0.1916 | 1.2188 | 0.1078 |
| Sub-perennial (adjusted values as modelled) |  | 0.6414 |  | 0.5188 |  |

## Determinants of implementation outcome

The Consolidated Framework of Implementation Research (CFIR) provides a guiding framework for systemic data collection from individuals who influence the implementation outcomes[[30](#_ENREF_30)]. It essentially includes five major domains, as outlined in Table S3. Each domain comprises of several factors or constructs that determine the outcome of an implementation.

**Table S3―Brief description of the CFIR domains and constructs, adapted to this project [**[30](#_ENREF_30)**,** [31](#_ENREF_31)**]**

| **Domain name** | **Domain definition** | **Construct names in each domain** |
| --- | --- | --- |
| **INNOVATION (renamed in the updated CFIR from INTERVENTION)[**[30](#_ENREF_30)**]** | The “thing” or intervention that is being implemented (such as, a proposed malaria chemoprevention strategy) | 1. Innovation Source 2. Innovation Evidence-Base 3. Innovation Relative Advantage 4. Innovation Adaptability 5. Innovation Trialability 6. Innovation Complexity 7. Innovation Design 8. Innovation Cost |
| **OUTER SETTING** | The setting that hosts the inner setting (such as, an epidemiological or clinical setting). Multiple outer settings or levels of outer setting may exist in a scenario. | 1. Policies & Laws 2. Local Conditions 3. Local Attitudes 4. Partnerships & Connections 5. Financing 6. Critical Incidents 7. External Pressure |
| **INNER SETTING** | The setting in which the innovation is actually implemented (such as, Expanded Program of Immunization (EPI) within a village level population) | 1. Structural Characteristics 2. Communications 3. Culture (e.g. Recipient-Centeredness, Deliverer-Centeredness) 4. Tension for Change 5. Available Resources (Funding, Space, Materials & Equipment) 6. Relative Priority 7. Incentive Systems 8. Access to Knowledge & Information |
| **INDIVIDUALS** | The roles and characteristics of individuals in the context of the implementation including the recipients of the innovation/intervention (such as, children receiving malaria chemoprevention, the EPI staff, national malaria control program officers) | 1. High-level Leaders 2. Mid-level Leaders 3. Opinion Leaders 4. Implementation Facilitators 5. Implementation Leads 6. Implementation Team Members 7. Other Implementation Support 8. Innovation Deliverers 9. Innovation Recipients |
| **IMPLEMENTATION PROCESS** | The strategies and list of activities that are applied to implement the innovation/intervention | 1. Teaming 2. Assessing Needs (of innovation deliverers and recipients) 3. Assessing Context 4. Planning 5. Tailoring Strategies 6. Engaging (innovation deliverers and recipients) 7. Doing 8. Reflecting & Evaluating (on both the implementation and the innovation) 9. Adapting |

# 2. Supplemental results

**
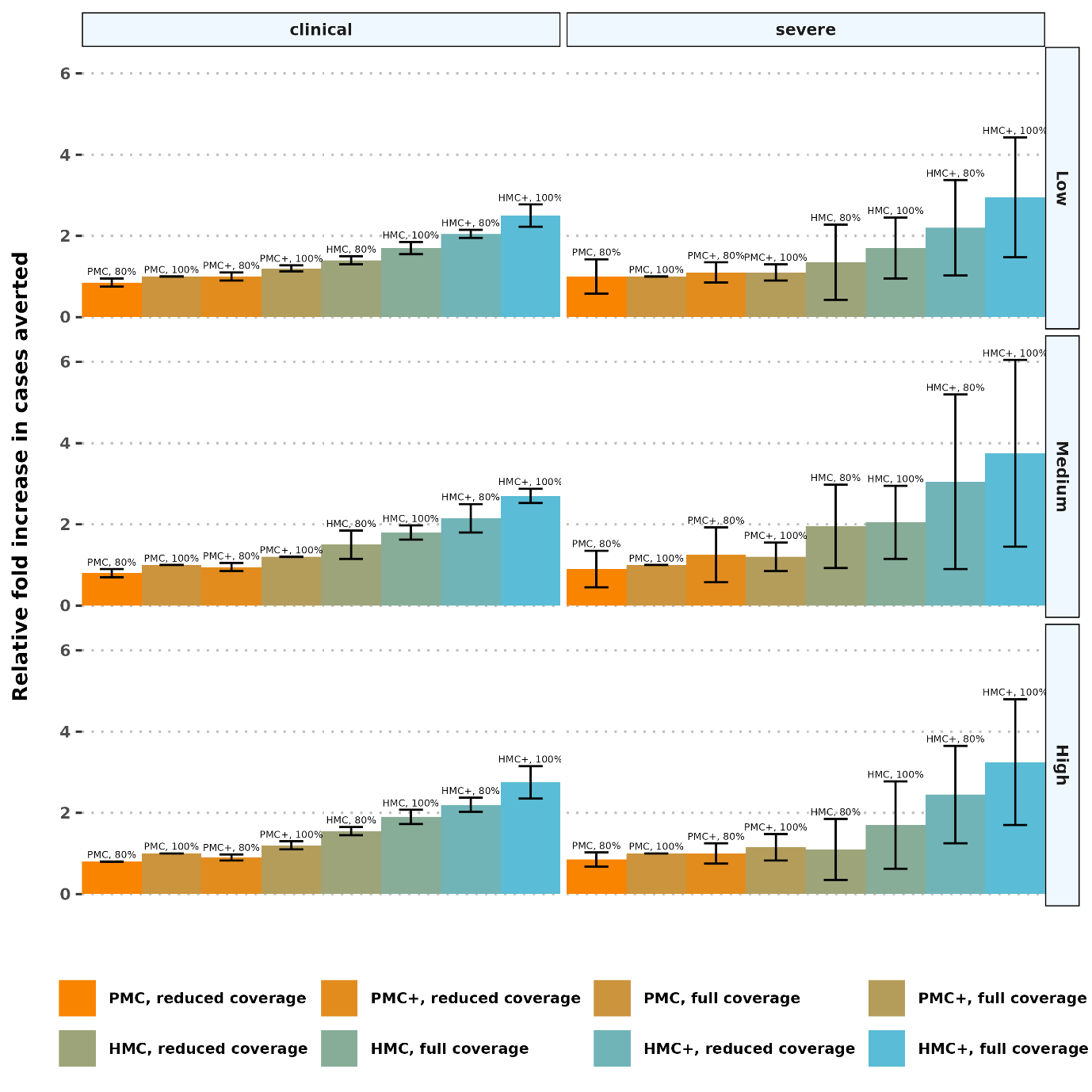
**

**Figure S1 Median relative fold increase in burden averted by the proposed dosing schedules (HMC or HMC+) compared to PMC alone, against all episodes of clinical and severe malaria in first three years of life. The efficacy or effectiveness values were compared to the full (100% in each dosing cycle) or reduced (80% in each dosing cycle) program coverage.** The relative fold increase in burden averted by PMC+ compared to PMC is also depicted for a reference to the added benefit achieved by only age-expansion. Varying health system strength is represented by the low (10%), medium (30%) and high (50%) probability of accessing case management in 14-days (aligned with Demographic Health Survey (DHS) definitions of 14-day treatment seeking for a fever) in medium to high transmission setting (*Pf*PR_2-10_ 30-39%, entomological inoculation rate 32). HMC: hybrid malaria chemoprevention. HMC+: age-expanded HMC; PMC: perennial malaria chemoprevention; PMC+: age-expanded perennial malaria chemoprevention; *Pf*PR_2-10 :_ *Plasmodium falciparum* prevalence in children age 2-10.

**
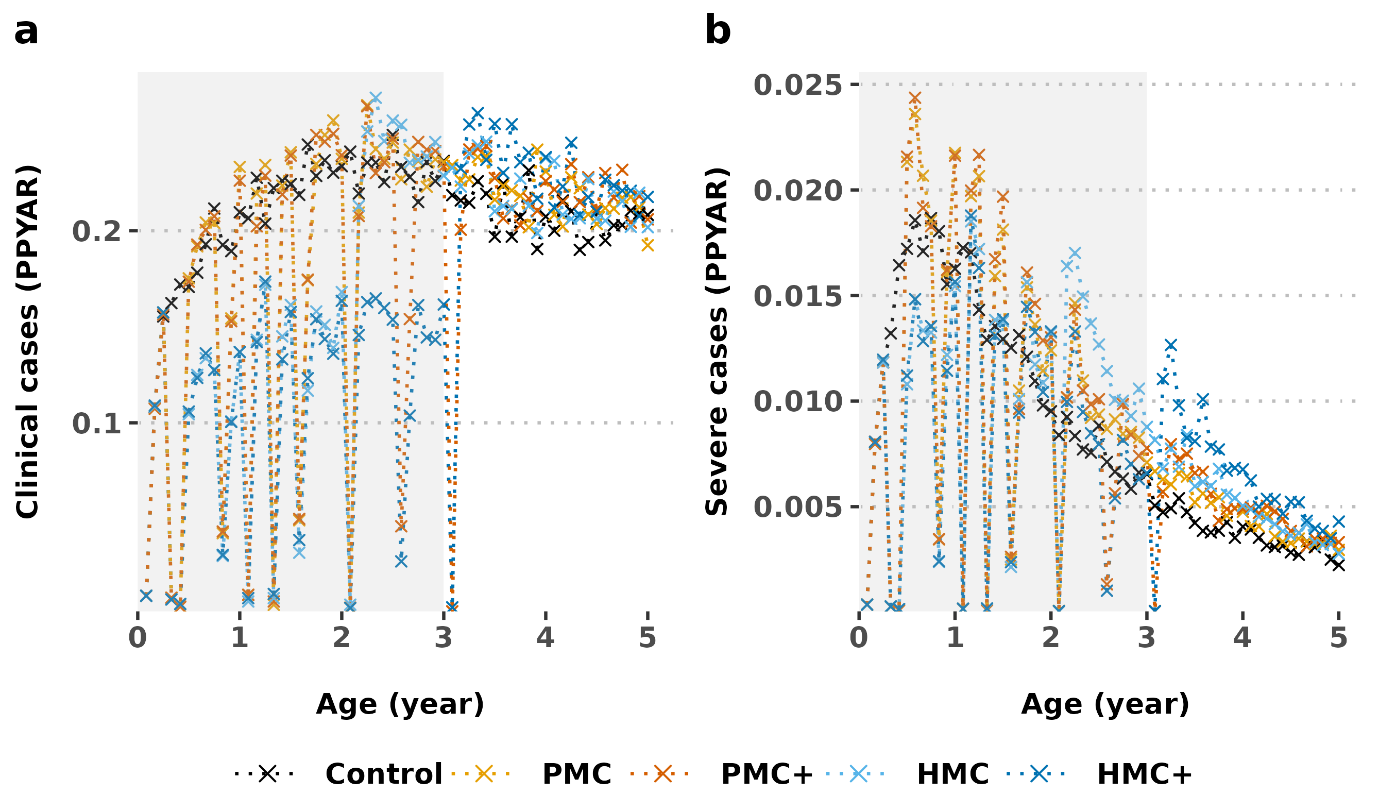
Figure S2 Age-pattern of clinical and severe malaria cases in intervention and follow-up ages.** The grey shading denotes the intervention cohort. Results depicted in settings with *Pf*PR_2-10_ 30–39%, (entomological inoculation rate 32, 30% probability of access to care (aligned with Demographic Health Survey (DHS) definitions of treatment seeking over 14-days ) and 100% program coverage in partially SP-resistance (quadruple mutant in *Pfdhfr* and *Pfdhps* genes that reduces prophylactic protection to 35 days from 42 days in a sensitive setting). HMC: hybrid malaria chemoprevention. HMC+: age-expanded HMC; PMC: perennial malaria chemoprevention; PMC+: age-expanded perennial malaria chemoprevention; PPYAR: Per person per year at risk; SP: sulphadoxine-pyrimethamine.

**Table S3**―**Potential determinants of implementation outcome as per Consolidated Framework for Implementation Research [**[30](#_ENREF_30)**,** [31](#_ENREF_31)**]**

| **Domain name** | **Constructs in the context of the proposed hybrid malaria chemoprevention** |
| --- | --- |
| **Innovation** | A proposed hybrid malaria chemoprevention delivery strategy (HMC, for children 03-24 months), and age-expanded HMC (HMC+, for children 03-36 months) for young children living in sub-perennial malaria transmission settings |
| **Outer setting** | 1. **Policies and laws:** malaria chemoprevention recommendation from the WHO, flexibility to adapting recommendations to the local context) 2. **Financing** (such as, The global Fund to Fight AIDS, Tuberculosis and Malaria - GFATM, Unitaid) 3. **Local conditions:** age-pattern of malaria and transmission intensity 4. **Partnerships and connections:** number and timings of Expanded Program of Immunization (EPI) touchpoints, possibility to recruit additional community health workers to deploy the seasonally-targeted chemoprevention during the high-risk rainy season |
| **Inner setting** | 1. **Structural Characteristics:** local EPI schedule, access to case management 2. **Relational Connections:** operational feasibility of delivery channels among EPI staff and venders 3. **Culture and Compatibility:** acceptability of intervention mix, adherence to schedule 4. **Available Resources:** program coverage, cost-effectiveness 5. **Mission Alignment:** drug resistance monitoring through genomic surveillance |
| **Individuals** | 1. **High-level leaders:** The WHO 2. **Mid-level Leaders:** national malaria control program 3. **Opinion Leaders:** sub-national health ministry, funding bodies 4. **Implementation leads:** district health facility, EPI, and other factors (media and communication) 5. **Innovation/intervention deliverers:** EPI staff, community health workers 6. **Recipients:** children under 36 months |

# References

1. WHO. Guidelines for malaria. Geneva: World Health Organization. 2023.

2. Sen S, Braunack-Mayer L, Kelly SL, Masserey T, Malinga J, Moehrle JJ, et al. Public health impact of current and proposed age-expanded perennial malaria chemoprevention: a modelling study. Sci Rep*.* 2025; 15**:**10488.

3. Smith T, Ross A, Maire N, Rogier C, Trape JF, Molineaux L. An epidemiologic model of the incidence of acute illness in *Plasmodium falciparum* malaria. Am J Trop Med Hyg*.* 2006; 75**:**56-62.

4. Smith T, Killeen GF, Maire N, Ross A, Molineaux L, Tediosi F, et al. Mathematical modeling of the impact of malaria vaccines on the clinical epidemiology and natural history of *Plasmodium falciparum* malaria: Overview. Am J Trop Med Hyg*.* 2006; 75**:**1-10.

5. Molineaux L, Diebner HH, Eichner M, Collins WE, Jeffery GM, Dietz K. *Plasmodium falciparum* parasitaemia described by a new mathematical model. Parasitology*.* 2001; 122**:**379-91.

6. Reiker T, Golumbeanu M, Shattock A, Burgert L, Smith TA, Filippi S, et al. Emulator-based Bayesian optimization for efficient multi-objective calibration of an individual-based model of malaria. Nat Commun*.* 2021; 12**:**7212.

7. WHO. Severe *falciparum* malaria. World Health Organization, Communicable Diseases Cluster. Trans R Soc Trop Med Hyg*.* 2000; 94 Suppl 1**:**S1-90.

8. WHO. World Malaria Report 2021. Geneva: World Health Organization. 2021.

9. Stone W, Gonçalves BP, Bousema T, Drakeley C. Assessing the infectious reservoir of *falciparum* malaria: past and future. Trends in Parasitology*.* 2015; 31**:**287-96.

10. Sinka ME, Pironon S, Massey NC, Longbottom J, Hemingway J, Moyes CL, et al. A new malaria vector in Africa: Predicting the expansion range of *Anopheles stephensi* and identifying the urban populations at risk. PNAS*.* 2020; 117**:**24900-8.

11. Smith T, Ross, A., Maire, N., Chitnis, N., Studer, A., Hardy, D., Brooks, A., Penny, M.,Tanner, M. Ensemble modeling of the likely public health impact of a pre-erythrocytic malaria vaccine. PLoS Med*.* 2012; 9**:**e1001157.

12. Golumbeanu M, Yang G-J, Camponovo F, Stuckey EM, Hamon N, Mondy M, et al. Leveraging mathematical models of disease dynamics and machine learning to improve development of novel malaria interventions. Infect Dis Poverty*.* 2022; 11**:**61.

13. Camponovo F, Jeandron A, Skrip LA, Golumbeanu M, Champagne C, Symons TL, et al. Malaria treatment for prevention: a modelling study of the impact of routine case management on malaria prevalence and burden. BMC Infect Dis*.* 2024; 24**:**1267.

14. Marsh K, Snow RW. Malaria transmission and morbidity. Parassitologia*.* 1999; 41**:**241-6.

15. Collins WE, Jeffery GM. A retrospective examination of the patterns of recrudescence in patients infected with *Plasmodium falciparum*. Am J Trop Med Hyg*.* 1999; 61**:**44-8.

16. Masserey T, Lee T, Golumbeanu M, Shattock AJ, Kelly SL, Hastings IM, et al. The influence of biological, epidemiological, and treatment factors on the establishment and spread of drug-resistant *Plasmodium falciparum*. eLife*.* 2022; 11**:**e77634.

17. Ross A, Maire N, Sicuri E, Smith T, Conteh L. Determinants of the cost-effectiveness of intermittent preventive treatment for malaria in infants and children. PLoS ONE*.* 2011; 6**:**e18391.

18. Ross A, Maire N, Molineaux L, Smith T. An epidemiologic model of severe morbidity and mortality caused by *Plasmodium falciparum*. Am J Trop Med Hyg*.* 2006; 75**:**63-73.

19. Stuckey EM, Smith T, Chitnis N. Seasonally dependent relationships between indicators of malaria transmission and disease provided by mathematical model simulations. PLoS Comput Biol*.* 2014; 10**:**e1003812.

20. Killeen GF, Ross A, Smith T. Infectiousness of malaria-endemic human populations to vectors. Am J Trop Med Hyg*.* 2006; 75**:**38-45.

21. Chitnis N, Hardy D, Smith T. A Periodically-Forced Mathematical Model for the Seasonal Dynamics of Malaria in Mosquitoes. Bull Math Biol*.* 2012; 74**:**1098-124.

22. Burgert L, Reiker T, Golumbeanu M, Möhrle JJ, Penny MA. Model-informed target product profiles of long-acting-injectables for use as seasonal malaria prevention. PLoS glob public health*.* 2022; 2**:**e0000211.

23. Pemberton-Ross P, Smith TA, Hodel EM, Kay K, Penny MA. Age-shifting in malaria incidence as a result of induced immunological deficit: a simulation study. Malar J*.* 2015; 14**:**287.

24. Tediosi F, Maire N, Smith T, Hutton G, Utzinger J, Ross A, et al. An approach to model the costs and effects of case management of *Plasmodium falciparum* malaria in sub-saharan Africa. Am J Trop Med Hyg*.* 2006; 75**:**90-103.

25. Penny MA, Verity R, Bever CA, Sauboin C, Galactionova K, Flasche S, et al. Public health impact and cost-effectiveness of the RTS,S/AS01 malaria vaccine: a systematic comparison of predictions from four mathematical models. Lancet*.* 2016; 387**:**367-75.

26. Wilcox RR. Chapter 6 - Some Multivariate Methods. In *Introduction to Robust Estimation and Hypothesis Testing (Fifth Edition).* Edited by Wilcox RR: *Academic Press*. 2022; 253-350

27. WHO. Malaria terminology 2021 update. Geneva: World Health Organization 2021.

28. Ashley EA, Yeka A. Seasonal malaria chemoprevention: closing the know-do gap. Lancet*.* 2020; 396**:**1778-9.

29. Macete E, Aide P, Aponte JJ, Sanz S, Mandomando I, Espasa M, et al. Intermittent preventive treatment for malaria control administered at the time of routine vaccinations in Mozambican infants: a randomized, placebo-controlled trial. J Infect Dis*.* 2006; 194**:**276-85.

30. Damschroder LJ, Reardon CM, Widerquist MAO, Lowery J. The updated Consolidated Framework for Implementation Research based on user feedback. Implement Sci*.* 2022; 17**:**75.

31. Damschroder LJ, Aron DC, Keith RE, Kirsh SR, Alexander JA, Lowery JC. Fostering implementation of health services research findings into practice: a consolidated framework for advancing implementation science. Implement Sci*.* 2009; 4**:**50.
